# Lower Mortality Risk Associated With Remdesivir + Dexamethasone Versus Dexamethasone Alone for the Treatment of Patients Hospitalized for COVID-19

**DOI:** 10.1093/cid/ciae477

**Published:** 2024-09-20

**Authors:** Essy Mozaffari, Aastha Chandak, Robert L Gottlieb, Chidinma Chima-Melton, Mark Berry, Thomas Oppelt, Jason F Okulicz, Alpesh N Amin, Tobias Welte, Paul E Sax, Andre C Kalil

**Affiliations:** Global Medical Affairs, Gilead Sciences, Foster City, California, USA; Evidence & Access, Certara, New York, New York, USA; Department of Internal Medicine, Baylor University Medical Center, Dallas, Texas, USA; Baylor Scott & White Heart and Vascular Hospital, Dallas, Texas, USA; Baylor Scott & White The Heart Hospital, Plano, Texas, USA; Baylor Scott & White Research Institute, Dallas, Texas, USA; Tele-ICU Inc, Los Angeles, California, USA; Real World Evidence, Gilead Sciences, Foster City, California, USA; US Medical Affairs, Gilead Sciences, Foster City, California, USA; Global Medical Affairs, Gilead Sciences, Foster City, California, USA; Division of Hospital Medicine & Palliative Medicine, Department of Medicine, University of California Irvine, California, USA; Department of Pulmonology and Infectious Diseases, Hannover Medical School, Hannover, Germany; Division of Infectious Diseases, Harvard Medical School, Brigham and Women's Hospital Boston, Massachusetts, USA; Department of Internal Medicine, Division of Infectious Diseases, University of Nebraska Medical Center, Omaha, Nebraska, USA

**Keywords:** COVID-19 guidelines, real-world data, mortality, propensity score matching, inverse probability of treatment weighting, data science, remdesivir

## Abstract

**Background:**

Treatment guidelines were developed early in the pandemic when much about coronavirus disease 2019 (COVID-19) was unknown. Given the evolution of severe acute respiratory syndrome coronavirus 2 (SARS-CoV-2), real-world data can provide clinicians with updated information. The objective of this analysis was to assess mortality risk in patients hospitalized for COVID-19 during the Omicron period receiving remdesivir + dexamethasone versus dexamethasone alone.

**Methods:**

A large, multicenter US hospital database was used to identify adult patients hospitalized with a primary discharge diagnosis of COVID-19 flagged as “present-on-admission” and treated with remdesivir + dexamethasone or dexamethasone alone between December 2021 and April 2023. Patients were matched using 1:1 propensity score matching and stratified by baseline oxygen requirements. Cox proportional hazards model was used to assess time to 14- and 28-day in-hospital all-cause mortality.

**Results:**

A total of 33 037 patients were matched, with most patients ≥65 years old (72%), White (78%), and non-Hispanic (84%). Remdesivir + dexamethasone was associated with lower mortality risk versus dexamethasone alone across all baseline oxygen requirements at 14-days (no supplemental oxygen charges: adjusted hazard ratio [95% confidence interval {CI}]: 0.79 [.72–.87], low flow oxygen: 0.70 [.64–.77], high flow oxygen/non-invasive ventilation: 0.69 [.62–.76], invasive mechanical ventilation/extracorporeal membrane oxygen (IMV/ECMO): 0.78 [.64–.94]), with similar results at 28-days.

**Conclusions:**

Remdesivir + dexamethasone was associated with a significant reduction in 14- and 28-day mortality compared to dexamethasone alone in patients hospitalized for COVID-19 across all levels of baseline respiratory support, including IMV/ECMO. However, the use of remdesivir + dexamethasone still has low clinical practice uptake. In addition, these data suggest a need to update the existing guidelines.

## Teaser

Remdesivir + dexamethasone was associated with reduced 14- and 28-day mortality versus dexamethasone alone in patients hospitalized for COVID-19 across all oxygen requirements supporting remdesivir use in invasive mechanical ventilation/extracorporeal membrane oxygen patients and suggesting the need to update existing guidelines.

**(See the Editorial Commentary by Lee on pages 72–3.)**

The World Health Organization (WHO) declared the severe acute respiratory syndrome coronavirus 2 (SARS-CoV-2) that causes coronavirus disease 2019 (COVID-19) a pandemic in March 2020 [1]. Over the 4 years since, guidelines have provided recommendations for use of antiviral and immunomodulatory (ie, corticosteroids) medications for COVID-19 management based on disease severity and evolving clinical evidence [2–4]. In 2024, WHO acknowledged COVID-19's continuing threat to lives and health systems [5].

In the early stages of the pandemic, the effectiveness of dexamethasone, an anti-inflammatory medication used to treat acute respiratory distress syndrome with mixed results [6, 7], was compared to usual care in patients with COVID-19 randomized during 19 March 2020 to 8 June 2020 (RECOVERY study, NCT04381936) [8]. This study led to the WHO recommendation for corticosteroids in the management of COVID-19 on 22 June 2020. The 28-day mortality in the dexamethasone group was lower compared to the usual care group (22.9% vs 25.7%). However, there was also the potential for detrimental effects of dexamethasone versus usual care in this study; the death rate was higher in patients receiving no oxygen at randomization treated with dexamethasone (17.8%) versus patients receiving usual care (14.0%). More recent research conducted since the RECOVERY study has also shown the potential for a detrimental effect of corticosteroid treatment in patients with low-severity COVID-19 [9, 10].

The Adaptive COVID-19 Treatment Trial (ACTT-1) (NCT04280705) evaluated the efficacy of the antiviral remdesivir in COVID-19 patients [11]. In this double-blind, randomized, placebo-controlled trial, remdesivir was superior to placebo in shortening time to recovery with a trend toward reduced 28-day mortality and a significant decrease in 14-day mortality, as well as both 28-day and 14-day mortality reductions seen in patients requiring low flow oxygen (LFO) at admission. In 2020, the US Food and Drug Administration (FDA) granted Emergency Use Authorization and then approval for remdesivir in adults and children hospitalized with COVID-19.

Current clinical guidelines for treatment of COVID-19 in hospitalized patients [2–4] include recommendations for use of remdesivir and/or dexamethasone (Table 1) and generally recommend dexamethasone without an antiviral only for patients on invasive mechanical ventilation/extracorporeal membrane oxygen (IMV/ECMO). Remdesivir + dexamethasone is recommended for most patients on supplemental LFO (typically ≤10–15 L/minute) or high flow oxygen/noninvasive mechanical ventilation (HFO/NIV) (typically >10–15 L/minute). The National Institutes of Health (NIH), Infectious Diseases Society of America (IDSA), and WHO Guidelines recommend use of remdesivir + dexamethasone in all patients on LFO. For patients on HFO/NIV, IDSA and WHO guidelines recommend use of remdesivir + dexamethasone. NIH guidelines recommend us of remdesivir + dexamethasone in immunocompromised patients or patients at high risk of progression to severe disease in those receiving HFO/NIV; other immunomodulatory agents (eg, baricitinib) are recommended for severe or critical COVID-19.

**Table 1.** Summary of Current COVID-19 Treatment Guidelines (as of June 2024) for Use of Dexamethasone and Remdesivir in Hospitalized Patients

Oxygen Requirements	Population	Treatment	NIH^a^	IDSA^b^	WHO^c^
No supplemental oxygen	Immunocompromised patients and/or patients at high risk of progression to severe disease	RDV	+^d^	+	+
		DEX	–	–	–
	All other patients	RDV	–	–	–
		DEX	–	–	–
Supplemental oxygen (LFO)	Patients who require minimal conventional oxygen	RDV	+^e,f^		
	All patients	RDV + DEX	+	+	+
Supplemental oxygen (HFO/NIV)	Immunocompromised patients and/or patients at high risk of progression to severe disease	RDV + DEX	+^g^	+	+
	All other patients	RDV	–	+	+
		DEX	+	+	+
IMV/ECMO	Immunocompromised patients and/or patients at high risk of progression to severe disease	RDV	+^h^	–	–
		DEX	+	+	+
	All other patients	RDV	–	–	–
		DEX	+	+	+

Abbreviations: COVID-19, coronavirus disease 2019; DEX, dexamethasone; HFO/NIV, high flow oxygen/non-invasive ventilation; IDSA, Infectious Diseases Society of America; IMV/ECMO, invasive mechanical ventilation/extracorporeal membrane oxygenation; LFO, low flow oxygen; NIH, National Institutes of Health; RDV, remdesivir; WHO, World Health Organization.

^a^Last updated on 29 February 2024.

^b^Last updated on 25 September 2020 for dexamethasone and 7 February 2022 for remdesivir.

^c^Last updated on 19 November 2023.

^d^Recommended for immunocompromised patients and other patients at high risk of progression to severe disease.

^e^Evidence suggests that the benefit of remdesivir is greatest when the drug is given early in the course of COVID-19 (eg, within 10 d of symptom onset).

^f^If these patients progress to requiring HFNC oxygen, NIV, MV, or ECMO, the full course of remdesivir should still be completed.

^g^Add remdesivir to immunocompromised patients, patients with ongoing viral replication, and patients who are within 10 d of onset of symptoms.

^h^Some NIH panel members would add remdesivir to immunomodulator therapy in patients recently placed on IMV/ECMO, who are immunocompromised, who have evidence of ongoing viral replication, or who are within 10 d of onset of symptoms.

**+** = recommended.

– = not recommended.

Real-world data (RWD) can be used to inform current COVID-19 treatment practice. For example, RWD demonstrated that remdesivir significantly reduced morality in patients hospitalized for COVID-19 not requiring supplemental oxygen [12]. Another study using RWD demonstrated that remdesivir significantly reduced mortality in patients hospitalized for COVID-19 requiring supplemental oxygen at admission, including those requiring HFO/NIV or IMV/ECMO [13].

In order to build on the findings from the early stages of COVID-19 and subsequent research over the COVID-19 era evolution including remdesivir studies using RWD, we undertook this RWD study to (1) assess in-hospital mortality in patients hospitalized for COVID-19 and treated with remdesivir + dexamethasone versus dexamethasone alone during the Omicron variant of concern (VOC) period stratified by oxygen requirements, and (2) to describe the use of remdesivir + dexamethasone and dexamethasone alone in routine clinical practice. This analysis was all inclusive of real-world practice, that is, included patients receiving remdesivir and/or dexamethasone independent of guideline recommendations.

## METHODS

### Study Design and Data Source

This was a retrospective, comparative effectiveness study using patient-level data from the PINC AI Healthcare Database representing ∼25% of yearly inpatient US hospitalizations and included demographics, disease state, diagnoses at admission and discharge, and billed services, among other patient-level information [14]. Less than 1% of patient records had missing information and, for key elements, such as demographics and diagnostic information, <0.01% of the patient records had missing data. In the PINC AI healthcare database, some institutions include oxygen as part of the room charge so it would not be charged for separately. As a result, we excluded hospitals that did not report any charges for oxygen supply or LFO device during the study period. This allowed us to only include those patients, wherein we could be confident that the absence of oxygen charges implied that the patient did not require supplement oxygen.

### Study Population

We included adult patients, hospitalized during the Omicron VOC period (December 2021 to April 2023) with a primary discharge diagnosis of COVID-19 (*International Classification of Diseases, 10th Revision, Clinical Modification* [ICD-10-CM] code U07.1) flagged as “present-on-admission.” The use of the COVID-19 diagnosis code (U07.1) has been previously validated in the PINC AI Healthcare Database [15]. Only the first hospitalization for COVID-19 during the study period was considered. Treatment groups consisted of patients who initiated remdesivir + dexamethasone or dexamethasone monotherapy in the first 2 days of hospitalization (baseline period). Patients in each treatment group were characterized by baseline supplemental oxygen requirements. Exclusion criteria included pregnancy, incomplete data, discharge or death within the first 2 days, transfer from hospice or another hospital, admission for an elective procedure, hospitals that did not report LFO charges (which may have been included as part of the hospital room services/charges), and initiation of COVID-19 treatments including baricitinib, tocilizumab (even if concordant with guidelines), or oral antivirals at baseline.

### Study Outcomes and Variables

Baseline covariates are defined in Supplementary Table 1 and include demographics, comorbidities, hospital characteristics, type of hospital ward on admission, COVID-19 treatments during baseline, admission month, and admission from a skilled nursing facility. Baseline supplemental oxygen requirements were characterized as no supplemental oxygen charges (NSOc), LFO, HFO/NIV, or IMV/ECMO. The follow-up period started the day after baseline until day 28 or discharge status of expired or hospice, transfer to another hospital, or addition of remdesivir after the first 2 days of hospitalization in the dexamethasone monotherapy group, whichever came first. All-cause inpatient mortality, defined as either “expired” or “hospice,” was assessed at 14 and 28 days after the baseline period. A sensitivity analysis was conducted considering only a discharge status of “expired” to define the outcome of interest.

### Statistical Analysis

All analyses were conducted overall and stratified by baseline supplemental oxygen requirements. Propensity scores (PSs) were estimated using separate logistic regression models for each baseline oxygen requirement category and included baseline covariates noted in Table 1. All covariates were retained in the model irrespective of their *P* value.

Using the derived PSs, distribution of underlying confounders in the two treatment groups was balanced using PS matching (PSM) as the primary analysis. To account for differences in hospital COVID-19 management practices, a 1:1 preferential within-hospital matching approach without replacement with a caliper distance of 0.2 times standard deviation of the logit of the PS was implemented. Patients receiving remdesivir + dexamethasone were matched to dexamethasone monotherapy patients in the same hospital within the specified caliper distance in the same age group (18–49, 50–64, ≥65 years), and admission month group (2–3 month blocks of admission month); unmatched patients were then matched to dexamethasone monotherapy patients in another remdesivir-using hospital of similar bed-size (<200, 200–499, ≥500 beds) within the specified caliper distance in the same age group and admission month group. Inverse probability of treatment weighting (IPTW) was performed as a sensitivity analysis [16]. In the IPTW approach, extreme PS scores <0.05 and >0.95 were trimmed.

Cox proportional hazards model was used to assess time to 14- and 28-day in-hospital all-cause mortality, and adjusted hazard ratios and 95% confidence intervals were derived. The models were adjusted for hospital-level cluster effects using a robust sandwich variance estimator, and key covariates of age, admission month, hospital admission ward (documented bed charges for intensive care unit [ICU]/step-down unit vs general ward), and time-varying covariates for treatments initiated after the baseline period such as baricitinib, tocilizumab, oral antivirals, or corticosteroids other than dexamethasone.

An additional PSM sensitivity analysis was performed to compare effectiveness of remdesivir + corticosteroid (including prednisone, prednisolone, methylprednisolone, hydrocortisone, and dexamethasone) versus corticosteroid monotherapy since other corticosteroids may also be used for COVID-19 treatment instead of dexamethasone.

## RESULTS

**Figure 1.**
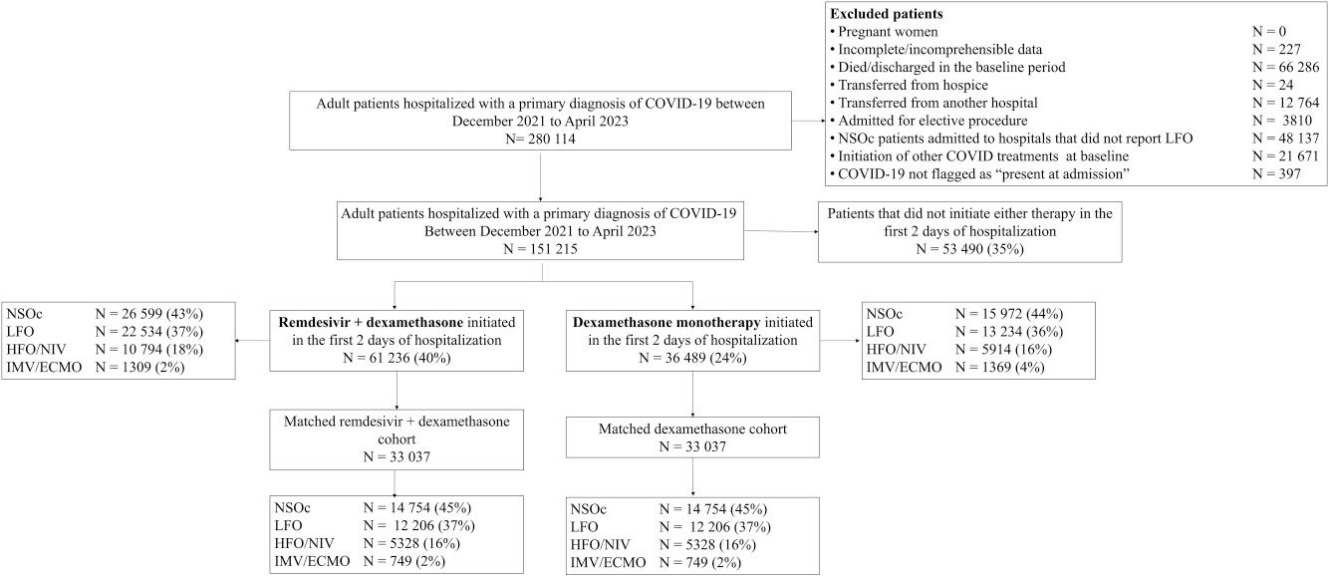
Study population for propensity score matching. Abbreviations: COVID-19, coronavirus disease 2019; HFO/NIV, high flow oxygen/non-invasive ventilation; IMV/ECMO, invasive mechanical ventilation/extracorporeal membrane oxygenation; LFO, low flow oxygen; NSOc, no supplemental oxygen charges.

Our study included 151 215 patients hospitalized for COVID-19 during the Omicron VOC period up to April 2023. A total of 61 236 (40%) initiated remdesivir + dexamethasone and 36 489 (24%) initiated dexamethasone monotherapy in the first 2 days of the hospitalization. Of the 36 489 patients receiving dexamethasone monotherapy, remdesivir was not initiated in 90% of the patients during the subsequent days of the hospitalization, while dexamethasone continued in 87% of the patients after the first 2 days of hospitalization. After 1:1 PSM, 33 037 remdesivir + dexamethasone patients were matched to 33 037 dexamethasone monotherapy patients (Figure 1).

Baseline demographics and hospital characteristics of the populations before and after PSM are shown in Table 2. Before matching, with respect to each individual demographic characteristic, most patients in the remdesivir + dexamethasone and dexamethasone monotherapy cohort, respectively, were ≥65 years (67%, 70%), White (78%, 77%), non-Hispanic (82%, 84%). Characteristics were well balanced after matching. Most patients did not receive supplemental oxygen at baseline (45%), the remaining patients received LFO (37%), HFO/NIV (16%), or IMV/ECMO (2%). Of the 15 972 patients that received dexamethasone monotherapy and did not require any supplemental oxygen at baseline, 11 814 (74%) patients did not require supplemental oxygen therapy throughout the hospitalization and 9641 (61%) continued receiving dexamethasone after the first 2 days in the hospital.

**Table 2.** Baseline Demographics and Hospital Characteristics of Patients Hospitalized for COVID-19 Between December 2021 and April 2023 (Omicron VOC Period), Before and After PSM

Characteristic		Before PSM			After PSM		
		Dexamethasone Monotherapy n = 36 489	Remdesivir + Dexamethasone n = 61 236	SMD	Dexamethasone Monotherapy n = 33 037	Remdesivir + Dexamethasone n = 33 037	SMD
Age group, y	18–49	3065 (8%)	6130 (10%)	0.09	2511 (8%)	2511 (8%)	0.00
	50–64	7845 (22%)	13 878 (23%)		6911 (21%)	6911 (21%)	
	≥65	25 579 (70%)	41 228 (67%)		23 615 (72%)	23 615 (72%)	
Gender	Female	18 469 (51%)	31 257 (51%)	0.01	16 749 (51%)	16 787 (51%)	0.00
Race	White	28 103 (77%)	47 614 (78%)	0.07	25 856 (78%)	25 820 (78%)	0.09
	Black	5258 (14%)	7596 (12%)		4371 (13%)	4465 (14%)	
	Asian	586 (2%)	1266 (2%)		538 (2%)	493 (2%)	
	Other	2542 (7%)	4760 (8%)		2272 (7%)	2259 (7%)	
Ethnicity	Hispanic	3169 (9%)	6795 (11%)	0.07	2945 (9%)	2868 (9%)	0.00
	Non-Hispanic	30 639 (84%)	50 516 (82%)		27 762 (84%)	27 820 (84%)	
	Unknown	2681 (7%)	3925 (6%)		2330 (7%)	2349 (7%)	
Primary Payor	Commercial	5179 (14%)	10 203 (17%)	0.10	4751 (14%)	4714 (14%)	0.04
	Medicare	26 334 (72%)	42 158 (69%)		23 976 (73%)	23 940 (72%)	
	Medicaid	2980 (8%)	5689 (9%)		2541 (8%)	2612 (8%)	
	Other	1996 (6%)	3186 (5%)		1769 (5%)	1771 (5%)	
Admission source	Transfer from SNF or ICF	1032 (3%)	1795 (3%)	0.01	942 (3%)	918 (3%)	0.00
Hospital size, no. of beds	≤100	3120 (9%)	5022 (8%)	0.14	2836 (9%)	2749 (8%)	0.06
	100–199	5859 (16%)	10 624 (17%)		5423 (16%)	5510 (17%)	
	200–299	7569 (21%)	12 412 (20%)		6949 (21%)	6834 (21%)	
	300–399	7358 (20%)	10 903 (18%)		6534 (20%)	6559 (20%)	
	400–499	4217 (12%)	6136 (10%)		3720 (11%)	3810 (12%)	
	≥500	8366 (23%)	16 139 (26%)		7575 (23%)	7575 (23%)	
Hospital location	Urban	31 236 (86%)	53 294 (87%)	0.04	28 367 (86%)	28 409 (86%)	0.00
	Rural	5253 (14%)	7942 (13%)		4670 (14%)	4628 (14%)	
Teaching hospital		14 341 (39%)	25 306 (41%)	0.04	12 969 (39%)	12 865 (39%)	0.01
Region	Midwest	8103 (22%)	13 421 (22%)	0.16	7445 (22%)	7348 (22%)	0.03
	Northeast	3838 (10%)	9495 (16%)		3637 (11%)	3721 (11%)	
	South	19 397 (53%)	28 860 (47%)		17 195 (52%)	17 062 (52%)	
	West	5151 (14%)	9460 (15%)		4760 (15%)	4906 (15%)	
Key comorbidities Hospital ward on admission	Obesity	10 810 (30%)	18 739 (31%)	0.02	9714 (29%)	9756 (30%)	0.03
	Chronic obstructive pulmonary disease	13 246 (36%)	23 359 (38%)	0.04	12 143 (37%)	12 084 (37%)	0.00
	Cardiovascular disease	32 210 (88%)	52 091 (85%)	0.09	28 974 (88%)	28 997 (88%)	0.00
	Diabetes	15 170 (42%)	23 498 (38%)	0.07	13 340 (40%)	13 310 (40%)	0.00
	Renal disease	12 950 (36%)	14 359 (23%)	0.27	10 524 (32%)	10 428 (32%)	0.01
	Immunocompromised condition	5999 (16%)	10 176 (17%)	0.01	5414 (16%)	5417 (16%)	0.00
	Cancer	2584 (7%)	4428 (7%)	0.01	2375 (7%)	2384 (7%)	0.00
	General ward	30 301 (83%)	50 596 (83%)	0.01	27 745 (84%)	28 063 (85%)	0.03
	Intensive care unit/Step-down unit	6188 (17%)	10 640 (17%)		5292 (16%)	4974 (15%)	
Diagnosis on admission	Sepsis	147 (0.4%)	189 (0.3%)	0.02	125 (0.4%)	121 (0.4%)	0.00
	Pneumonia	2573 (7%)	4063 (7%)	0.02	2213 (7%)	2186 (7%)	0.00
Other treatments at baseline	Anticoagulants	27 322 (75%)	49 560 (81%)	0.15	25 592 (78%)	25 546 (77%)	0.00
	Convalescent plasma	27 (0.1%)	69 (0.1%)	0.01	23 (0.1%)	24 (0.1%)	0.00
	Corticosteroids other than dexamethasone	5035 (14%)	9252 (15%)	0.04	4602 (14%)	4514 (14%)	0.01
Baseline supplemental oxygen requirements	NSOc	15 972 (44%)	26 599 (43%)	0.13	14 754 (45%)	14 754 (45%)	0.00
	LFO	13 234 (36%)	22 534 (37%)		12 206 (37%)	12 206 (37%)	
	HFO/NIV	5914 (16%)	10 794 (18%)		5328 (16%)	5328 (16%)	
	IMV/ECMO	1369 (4%)	1309 (2%)		749 (2%)	749 (2%)	

Abbreviations: COVID-19, coronavirus disease 2019; HFO/NIV, high flow oxygen/non-invasive ventilation; ICF, intermediate care facility; IMV/ECMO, invasive mechanical ventilation/extracorporeal membrane oxygenation; IPTW, inverse probability of treatment weighting; LFO, low flow oxygen; no., number; NSOc, no supplemental oxygen charges; PSM, propensity score matching; SMD, standardized mean difference; SNF, skilled nursing facility; VOC, variant of concern; y, years.

Baseline demographics and hospital characteristics of the populations before and after IPTW are shown in Supplementary Table 2. After IPTW, most patients were ≥65 years (68%), White (78%), and non-Hispanic (83%). Most patients did not receive supplemental oxygen at baseline (44%); the remaining patients received LFO (37%), HFO/NIV (17%), and IMV/ECMO (3%).

Mortality rates in the PS matched cohort were significantly lower for remdesivir + dexamethasone versus dexamethasone monotherapy across all baseline supplemental oxygen groups. For the NSOc group, 5.6% and 7.2% of remdesivir + dexamethasone patients died within 14 and 28 days, respectively, compared to 6.1% and 7.7% of dexamethasone monotherapy patients. For patients receiving LFO, 6.1% and 8.1% of remdesivir + dexamethasone patients died within 14 and 28 days, respectively, compared to 7.7% and 9.7% of dexamethasone monotherapy patients. For patients receiving HFO/NIV, 12.7% and 17.6% of remdesivir + dexamethasone patients died within 14 and 28 days, respectively, compared to 15.7% and 20.7% of dexamethasone monotherapy patients. For patients receiving IMV/ECMO, 23.5% and 32.7% of remdesivir + dexamethasone patients died within 14 and 28 days, respectively, compared to 27.1% and 35.4% of dexamethasone monotherapy patients.

**Figure 2.**
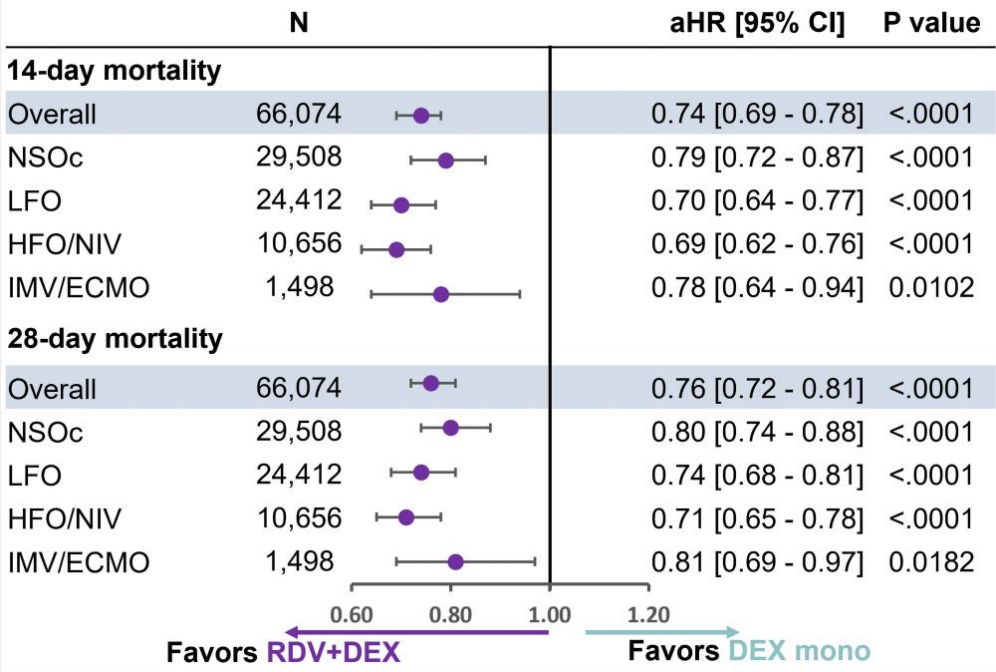
14- and 28-day mortality in patients hospitalized for COVID-19 receiving RDV + DEX or DEX monotherapy by supplemental oxygen requirements: 1:1 propensity score matching without replacement. Estimates adjusted for age, admission month, hospital ward on admission (ICU vs general ward) and time-varying treatment with other COVID-19 medications (baricitinib, tocilizumab, oral antivirals). Abbreviations: aHR, adjusted hazard ratio; CI, confidence interval; COVID-19, coronavirus disease 2019; DEX, dexamethasone; HFO/NIV, high flow oxygen/noninvasive ventilation; ICU, intensive care unit; IMV/ECMO, invasive mechanical ventilation/extracorporeal membrane oxygenation; LFO, low flow oxygen; mono, monotherapy; NSOc, no supplemental oxygen charges; RDV, remdesivir.

Further, in the PS matched cohort (Figure 2), remdesivir + dexamethasone was associated with a lower mortality risk versus dexamethasone monotherapy overall and across all baseline oxygen requirements at 14 days (Overall: adjusted hazard ratio [95% CI]: 0.74 [.69–.78], NSOc: 0.79 [.72–.87], LFO: 0.70 [.64–.77], HFO/NIV: 0.69 [.62–.76], IMV/ECMO: 0.78 [.64–.94]); and at 28 days (Overall: 0.76 [.72–.81], NSOc: 0.80 [.74–.88], LFO: 0.74 [.68–.81], HFO/NIV: 0.71 [.65–.78], and IMV/ECMO: 0.81 [.69–.97]).

Similarly, using IPTW as a sensitivity analysis (Supplementary Figure 1), remdesivir + dexamethasone was associated with a lower mortality risk versus dexamethasone monotherapy across all baseline oxygen requirements at 14 days (NSOc: 0.80 [.73–.87], LFO: 0.70 [.64–.76], HFO/NIV: 0.73 [.66–.80], IMV/ECMO: 0.85 [.73–.98]); and at 28 days (NSOc: 0.81 [.75–.88], LFO: 0.73 [.67–.79], HFO/NIV: 0.74 [.68–.81], IMV/ECMO: 0.86 [.76–.98]). Results were consistent in the sensitivity analyses considering a discharge status of “expired” (Supplementary Figure 2) and also comparing treatment with remdesivir + corticosteroid to corticosteroid monotherapy (Supplementary Figure 3).

We also compared guideline recommendations to the real-world use of remdesivir + dexamethasone and dexamethasone monotherapy in clinical practice observed in this cohort. In patients hospitalized with a primary diagnosis of COVID-19 from December 2021 to April 2023, 35% did not receive remdesivir or dexamethasone in the first 2 days of hospitalization despite guidelines that recommend remdesivir + dexamethasone in the majority of hospitalized patients. Of the patients not receiving supplemental oxygen at baseline (n = 42 571), 62.5% received remdesivir + dexamethasone and 37.5% received dexamethasone monotherapy, despite the recommendation against dexamethasone use in these patients. The majority of patients not receiving supplemental oxygen at baseline that received dexamethasone monotherapy did not require supplemental oxygen throughout their hospitalization but continued to receive dexamethasone. In patients with LFO at baseline (n = 35 768), 37% should have also received remdesivir according to guidelines. Guidelines recommend the use of remdesivir in hospitalized patients with NSOc, LFO, HFO, or NIV; however, of the 36 489 patients included in the study who received dexamethasone monotherapy at hospital admission, 90% (n = 32 980) did not receive remdesivir during subsequent days in the hospital.

## DISCUSSION

COVID-19 remains a threat to lives, especially in patients who are immunocompromised, elderly, or have comorbidities, and a burden to health systems worldwide [5] with more than 75 000 deaths due to COVID-19 in the United States in 2023 [17]. Therefore, there is a continuing need to improve our treatment approach and optimize our choice of treatment options based upon the evolving evidence across the endemic COVID-19 era.

Much of the more commonly cited evidence regarding effectiveness of COVID-19 therapies and clinical treatment decisions is based on data obtained in the early stages of the pandemic even though the evidence has evolved considerably. NIH guidelines, updated for the final time on 29 February 2024, suggest that there is insufficient evidence to recommend for or against remdesivir use in patients who require IMV/ECMO. However, in this final update, some NIH panelists recommended adding remdesivir to immunomodulator therapy in patients recently placed on IMV/ECMO, who are immunocompromised, have evidence of ongoing viral replication, or are within 10 days of COVID-19 symptom onset [2]. Retrospective analyses utilizing RWD can help clarify and support updates to COVID-19 treatment guidelines.

Our study, using 2 well-established methods in comparative effectiveness research, PSM and then IPTW as a sensitivity analysis [16], illustrates the utility of RWD to support appropriate therapy developed by previous randomized controlled trials (RCTs) and inform guidelines, as repeating multiple RCTs may not be feasible in the current endemic COVID-19 era. Both analytical approaches demonstrated that remdesivir + dexamethasone was associated with significantly lower mortality risk in patients hospitalized for COVID-19 at 14 days and 28 days versus dexamethasone alone across all baseline oxygen requirements, including a significant 19% reduction in 28-day mortality in IMV/ECMO patients who received dexamethasone + remdesivir compared to dexamethasone alone among patients who did not receive baricitinib or tocilizumab. This supports the use of remdesivir + dexamethasone in this patient population and further supports the growing evidence that viral replication may persist late in the course of the disease even in patients who require IMV/ECMO and are not immunocompromised. Our study excluded patients receiving baricitinib or tocilizumab in the first 2 days of hospitalization in order to assess the impact of two treatments independently of other COVID-19 treatments. Thus, the findings of this study are not applicable to patients that could be receiving either of these therapies. Additionally, the utilization of baricitinib and tocilizumab is only applicable to <5% because of the lower incidence of patients critically ill due to COVID-19 in more recent times. Notably, the ACTT-2 trial included foundational remdesivir for all patients, with only a minority of patients receiving glucocorticoids [18], whereas the COV-Barrier trial enrolled patients predominantly treated with glucocorticoids at baseline, with a minority receiving remdesivir [19]. The role of combination immunomodulatory agents is beyond the scope of our study.

Evolving evidence generated during later stages of the pandemic shows a lack of benefit, or even harm of dexamethasone monotherapy in patients not receiving oxygen [9, 10]. Remdesivir + dexamethasone was associated with a significant survival benefit as compared to dexamethasone monotherapy in patients not receiving supplemental oxygen. This new finding strongly suggests that viral clearance delay is likely the reason for worse survival outcomes with dexamethasone in the early course of COVID-19 (before patients require supplemental oxygen), and that this may be mitigated by the antiviral activity of remdesivir. Nevertheless, this finding should not be used to encourage the use of glucocorticoids in patients with COVID-19 without hypoxemia in the absence of a compelling indication for steroids for a reason unrelated to COVID-19. A natural extrapolation of these data, however, is that when there is chronic preexisting use of glucocorticoids, the addition of remdesivir may attenuate the risk conferred by glucocorticoids in the COVID-19 population without hypoxemia. This finding of significantly lower mortality risk across all respiratory support levels in patients receiving remdesivir + dexamethasone versus dexamethasone alone matches not only the expected biologic course of the infection (faster viral clearance leads to short disease duration and improved survival) but is also highly consistent with all infectious disease evidence in which steroids’ survival benefits are only present when given together with antimicrobials in severe infections such as *Streptococcus pneumoniae* meningitis [20], *Pneumocystis jirovecii* [21], and septic shock [22] where steroid monotherapy is never recommended. Furthermore, our study suggests that it is important to target the SARs-CoV-2 virus directly, especially in this endemic era when hospitalized patients with COVID-19 are likely to have significant comorbidities and be at higher risk of developing severe disease.

Our findings suggest that, for many patients, guidelines for the use remdesivir + dexamethasone are not being adhered to in clinical practice. NIH, IDSA, and WHO Guidelines recommend use of remdesivir + dexamethasone in all patients on LFO. For patients on HFO/NIV, IDSA and WHO guidelines recommend use of remdesivir + dexamethasone. NIH guidelines recommend use of remdesivir + dexamethasone in immunocompromised patients or patients at high risk of progression to severe disease in those receiving HFO/NIV. Many patients are being treated with dexamethasone monotherapy across the range of supplemental oxygen support, including patients with no oxygen support requirements, which goes against current guideline recommendations. It should also be noted that NIH guidelines recommend remdesivir for immunocompromised patients or patients at risk of progression to severe disease, across all levels of oxygen support [2]. The reasons for lack of adherence to the guidelines is not clear and warrants further research. However, this study suggests the potential impact in terms of in-hospital mortality when guidelines are not followed.

The strengths of this study include the large population from a multicenter administrative database. The study applied 2 well-established methods, PSM and IPTW, to balance inherently different groups due to confounding by indication. Consistent results were obtained with the 2 methods indicating association of remdesivir + dexamethasone with a significantly lower mortality risk at 14 and 28 days versus dexamethasone alone in patients with COVID-19 across all baseline oxygen requirements. This and future observational research can complement and build on the findings from RCTs and subsequent research over the evolution of the COVID-19 era. Future studies to corroborate and extend these findings will require a fit-for-purpose database with adequate sample size to power the study, high-quality data, and rigorous methods to address bias [16].

As with any observational study, there are limitations including the potential for residual confounding due to imbalances in unmeasured variables between the treatment groups even after PSM, which was minimized by matching patients according to the PS, age-group, admission month, and hospital. Also, data on time of symptom onset or time since first positive COVID-19 test were not available in this database. However, the analyses were stratified by baseline oxygen requirements as a surrogate for disease severity and benefits of remdesivir + dexamethasone were observed across different disease severities. A homogenous cohort was ensured by restricting the study to patients admitted to the hospital with a primary discharge diagnosis of COVID-19 flagged as “present on admission.” Immortal-time bias was addressed by including both eligibility criteria and treatment assignment at time-zero in both treatment arms, as well as by the landmark survival analysis. No vaccination data were available in the database; however, majority of the population was vaccinated during this study's Omicron VOC period. Baseline supplemental oxygen requirements were assessed via billing charges for supplemental oxygen. As some hospitals may include supplemental oxygen charges with room charges, patients from hospitals that did not report any charges for LFO were not include in the NSOc group to ensure that data were from hospitals that uniformly report supplemental oxygen requirements. Finally, data on antiviral use or any other treatment administered prior to hospitalization were unavailable, which may have led to residual confounding.

Findings from this research suggest that more effort is needed to update guidelines recommending the use of remdesivir + dexamethasone in patients requiring supplemental oxygen according to best practice guidelines for oxygen use, and the strong clinical rationale to avoid dexamethasone monotherapy in all levels of respiratory support, especially in those not receiving supplemental oxygen. Furthermore, these findings reinforce the recent evidence from RWD supporting the use of antiviral treatment for hospitalized COVID-19 patients across all oxygen levels [12, 13] and the need to update and clarify clinical treatment guidelines. The current ambiguities in COVID-19 guidelines may be, in part, leading to underutilization of antiviral treatment in situations where RWD suggests it reduces mortality risk, despite underpowered data earlier in the pandemic (eg, patients on IMV/ECMO).

In summary, our study highlights that the addition of remdesivir to dexamethasone is associated with a significant survival benefit compared to dexamethasone without remdesivir use. This finding is observed in patients without supplemental oxygen requirements (a group for whom dexamethasone usage is contraindicated unless for a pre-existing condition or for treatment of a non-COVID-19 condition) as well as in patients with hypoxemia across the spectrum of oxygen support requirements, for whom the addition of remdesivir further improves outcomes.

## Supplementary Data

Supplementary materials are available at *Clinical Infectious Diseases* online. Consisting of data provided by the authors to benefit the reader, the posted materials are not copyedited and are the sole responsibility of the authors, so questions or comments should be addressed to the corresponding author.

## Supplementary Material

ciae477_Supplementary_Data

## Notes

***Author contributions.*** All authors contributed substantially to the manuscript and agreed to the final submitted version.

***Financial support.*** This work was funded by Gilead Sciences Inc.
