# Supplementary material for: Lower Mortality Risk Associated With Remdesivir + Dexamethasone Versus Dexamethasone Alone for the Treatment of Patients Hospitalized for COVID-19
Source: Clin Infect Dis. 2024 Sep 20;80(1):63–71. doi: 10.1093/cid/ciae477 (PMC11797391; doi:10.1093/cid/ciae477)
Supplement: ciae477_Supplementary_Data [file ciae477_supplementary_data.docx]

Lower mortality risk associated with remdesivir + dexamethasone versus dexamethasone alone for the treatment of patients hospitalized for COVID-19

Essy Mozaffari^1^, Aastha Chandak^2^, Robert L. Gottlieb^3,4,5,6^, Chidinma Chima-Melton^7^, Mark Berry^1^, Thomas Oppelt^1^, Jason F. Okulicz^1^, Alpesh N. Amin^8^, Tobias Welte^9*^, Paul E. Sax^10^, Andre C. Kalil^11^

^1^ Gilead Sciences, Foster City, California, USA

^2^ Certara, New York, New York, USA

^3^ Baylor University Medical Center, Dallas, Texas, USA

^4^ Baylor Scott & White Heart and Vascular Hospital, Dallas, Texas, USA

^5^ Baylor Scott & White The Heart Hospital, Plano, Texas, USA

^6^ Baylor Scott & White Research Institute, Dallas, Texas, USA

^7^ University of California, Los Angeles, California, USA

^8^ University of California Irvine, California, USA

^9^Klinik für Innere Medizin III, Universitätsklinikum des Saarlandes, Saarland University, Homburg/Saar, Germany

^10^Harvard Medical School, Brigham and Women’s Hospital Boston, Massachusetts, USA

^11^ University of Nebraska Medical Center, Omaha, Nebraska, USA

*Dr. Tobias Welte is deceased.

# Supplementary Material

**Table S1. Definitions of key study variables**

| Key Study Variables | | Definitions |
| --- | --- | --- |
| Remdesivir treatment |  | Billing charges for treatment at baseline: Remdesivir;  ICD-10 procedure codes: XW033E5, XW043E5 |
| Key Comorbidities | Obesity | ICD-10-CM diagnosis codes: E66, Z6825-Z6845 |
|  | COPD | ICD-10-CM diagnosis codes: I278, I279, J40, J41, J42, J43, J44, J45, J46, J47, J60, J61, J62, J63, J64, J65, J66, J67, J684, J701, J703 |
|  | Cardiovascular disease (including hypertension) | ICD-10-CM diagnosis codes: I00-I99 |
|  | Diabetes | ICD-10-CM diagnosis codes: E10-E14 |
|  | Renal disease | ICD-10-CM diagnosis codes: I120, I131, N032, N033, N034, N035, N036, N037, N052, N053, N054, N055, N056, N057, N18, N19, N250, Z490, Z491, Z492, Z940, Z992 |
|  | Cancer | ICD-10-CM diagnosis codes: C00-C96 |
|  | Immunocompromised condition | ICD-10-CM code for cancer (C00-C96), transplant (Z94.x), hematologic malignancies (C81.x, C82.x, C83.x, C84.x, C85.x, C88.x, C90.x, C91.x, C92.x, C93.x, C94.x, C95.x, C96.x) , primary immunodeficiencies (D80.x, D81.x, D82.x, D83.x, D84.x, G11.3, E70.330, D71.x, D70.x), asplenia (Q89.01, Z90.81), toxic effects of antineoplastics (T45.1x), bone marrow failure/aplastic anemia (D61.x), severe combined immunodeficiencies (D80.x, D81.x, D82.x, D83.x, D84.x, D86.x, D89.0, D89.1, D89.2, D89.3 , D89.4x, D89.81, D89.82, D89.89, D89.9), HIV (B20), patients with chronic graft-versus-host disease or who are taking immunosuppressive medications for another indication (Z89.8x, Z79.52, Z79.61, Z79.62x, Z79.63x, Z79.64, Z79.69, Z79.810, Z79.811, Z79.818) |
| Supplemental oxygen requirements | IMV/ECMO | Billing charges for devices: invasive mechanical ventilation, tracheostomy, endotracheal tube, intubation, extracorporeal membrane oxygenation |
|  | HFO/NIV | Billing charges for devices: negative-pressure ventilation, positive-pressure ventilation, CPAP, BiPAP, high flow system via nasal cannula, venturi face mask, rebreather, non-rebreather mask, positive expiratory pressure |
|  | LFO | Billing charges for devices/oxygen supply: Simple face mask, oxygen pendant, low flow system via nasal cannula, oxygen supply |
|  | NSO | No billing charges for IMV/ECMO, HFO/NIV, or LFO at baseline. |
| Admitting Diagnosis | Sepsis | ICD-10-CM diagnosis codes: A021, A327, A40, A41, A427, A5486, B377, R6520, R6521, T8144X, |
|  | Pneumonia | ICD-10-CM diagnosis codes: J12, J13, J14, J15, J16, J17, J18, A481, B250, A3701, A3711, A3781, A3791, A221, B440, B7781, J1000, J1001, J1008, J1100, J1108 |
| Other treatments | Anticoagulants | Billing charges for treatment: Treatments used at baseline: apixaban, argatroban, desirudin, lepirudin, dabigatran, danaparoid, edoxaban, tinzaparin, heparin (excluding use of heparin flush), ardeparin, bivalirudin |
|  | Corticosteroids | Billing charges for treatment: prednisone, prednisolone, methylprednisolone, hydrocortisone, dexamethasone, |
|  | Convalescent plasma | Billing charges for treatment at baseline: convalescent plasma; ICD-10 procedure codes: XW13325, XW14325 |
|  | Baricitinib | Billing charges for treatment: Baricitinib; ICD-10 procedure codes: XW0DXM6, XW0H7M6, XW0G7M6 |
|  | Tocilizumab | Billing charges for treatment: Tocilizumab; ICD-10 procedure codes: XW033H5, XW043H5 |
|  | Oral antivirals | Billing charges for treatment: nirmatrelvir ritonavir, molnupiravir |

**Table S2**. Baseline characteristics (before and after IPTW)

| **Characteristic** | | **Before Matching or IPTW** | | | **After IPTW** | | |
| --- | --- | --- | --- | --- | --- | --- | --- |
|  |  | **Dexamethasone monotherapy**  **n = 36 489** | **Remdesivir + dexamethasone n = 61 236** | **SMD** | **Dexamethasone monotherapy**  **n = 97 780** | **Remdesivir + dexamethasone n = 97 697** | **SMD** |
| **Age group, y** | 18-49 | 3065 (8%) | 6130 (10%) | 0.09 | 9205 (9%) | 9192 (9%) | 0.00 |
|  | 50-64 | 7845 (22%) | 13 878 (23%) |  | 21 792 (22%) | 21 732 (22%) |  |
|  | 65+ | 25 579 (70%) | 41 228 (67%) |  | 66 783 (68%) | 66 773 (68%) |  |
| **Gender** | Female | 18 469 (51%) | 31 257 (51%) | 0.01 | 49 708 (51%) | 49 685 (51%) | 0.00 |
| **Race** | White | 28 103 (77%) | 47 614 (78%) | 0.07 | 75 851 (78%) | 75 748 (78%) | 0.00 |
|  | Black | 5258 (14%) | 7596 (12%) |  | 12 782 (13%) | 12 804 (13%) |  |
|  | Asian | 586 (2%) | 1266 (2%) |  | 1846 (2%) | 1851 (2%) |  |
|  | Other | 2542 (7%) | 4760 (8%) |  | 7302 (8%) | 7294 (8%) |  |
| **Ethnicity** | Hispanic | 3169 (9%) | 6795 (11%) | 0.06 | 9869 (10%) | 9938 (10%) | 0.00 |
|  | Non-Hispanic | 30 639 (84%) | 50 516 (82%) |  | 81 282 (83%) | 81 149 (83%) |  |
|  | Unknown | 2681 (7%) | 3925 (6%) |  | 6629 (7%) | 6610 (7%) |  |
| **Primary Payor** | Commercial | 5179 (14%) | 10 203 (17%) | 0.10 | 15 349 (16%) | 15 365 (16%) | 0.00 |
|  | Medicare | 26 334 (72%) | 42 158 (69%) |  | 68 501 (70%) | 68 457 (70%) |  |
|  | Medicaid | 2980 (8%) | 5689 (9%) |  | 8717 (9%) | 8684 (9%) |  |
|  | Other | 1996 (6%) | 3186 (5%) |  | 5213 (5%) | 5191 (5%) |  |
| **Admission Source** | Transfer from SNF or ICF | 1032 (3%) | 1795 (3%) | 0.01 | 2819 (3%) | 2824 (3%) | 0.04 |
| **Hospital size, no. of beds** | <100 | 3120 (9%) | 5022 (8%) | 0.14 | 8209 (8%) | 8209 (8%) | 0.04 |
|  | 100-199 | 5859 (16%) | 10 624 (17%) |  | 16 502 (17%) | 16 502 (17%) |  |
|  | 200-299 | 7569 (21%) | 12 412 (20%) |  | 19 951 (20%) | 19 951 (20%) |  |
|  | 300-399 | 7358 (20%) | 10 903 (18%) |  | 18 164 (19%) | 18 164 (19%) |  |
|  | 400-499 | 4217 (12%) | 6136 (10%) |  | 10 346 (11%) | 10 346 (11%) |  |
|  | 500+ | 8366 (23%) | 16 139 (26%) |  | 24 525 (25%) | 24 525 (25%) |  |
| **Key comorbidities** | Obesity | 10 810 (30%) | 18 739 (31%) | 0.02 | 29 607 (30%) | 29 574 (30%) | 0.00 |
|  | Chronic obstructive pulmonary disease | 13246 (36%) | 23 359 (38%) | 0.04 | 36 715 (38%) | 36 641 (38%) | 0.00 |
|  | Cardiovascular disease | 32 210 (88%) | 52 091 (85%) | 0.09 | 84 373 (86%) | 84 276 (86%) | 0.00 |
|  | Diabetes | 15 170 (42%) | 23 498 (38%) | 0.07 | 38 586 (40%) | 38 594 (40%) | 0.00 |
|  | Renal disease | 12 950 (36%) | 14 359 (23%) | 0.27 | 27 372 (28%) | 27 290 (28%) | 0.00 |
|  | Immunocompromised condition | 5999 (16%) | 10 176 (17%) | 0.01 | 16 280 (17%) | 16 209 (17%) | 0.00 |
|  | Cancer | 2584 (7%) | 4428 (7%) | 0.01 | 7068 (7%) | 7025 (7%) | 0.00 |
| **Hospital ward on admission** | General ward | 30 301 (83%) | 50 596 (83%) | 0.01 | 81 150 (83%) | 80 904 (83%) | 0.01 |
|  | ICU/step-down unit | 6188 (17%) | 10 640 (17%) |  | 16 631 (17%) | 16 794 (17%) |  |
| **Other treatments at baseline** | Anticoagulants | 27 322 (75%) | 49 560 (81%) | 0.15 | 77 063 (79%) | 76 958 (79%) | 0.00 |
|  | Convalescent plasma | 27 (0.1%) | 69 (0.1%) | 0.01 | 92 (0.1%) | 96 (0.1%) | 0.00 |
|  | Other corticosteroids | 5035 (14%) | 9252 (15%) | 0.04 | 14 371 (15%) | 14 326 (15%) | 0.00 |
| **Baseline supplemental oxygen requirements** | NSOc | 15 972 (44%) | 26 599 (43%) | 0.13 | 42 634 (44%) | 42 550 (44%) | 0.00 |
|  | LFO | 13 234 (36%) | 22 534 (37%) |  | 35 758 (37%) | 35 760 (37%) |  |
|  | HFO/NIV | 5914 (16%) | 10 794 (18%) |  | 16 715 (17%) | 16 705 (17%) |  |
|  | IMV/ECMO | 1369 (4%) | 1309 (2%) |  | 2673 (3%) | 2682 (3%) |  |
| Abbreviations: DEX, dexamethasone; HFO/NIV, high flow oxygen/non-invasive ventilation; ICF, intermediate care facility; ICU, intensive care unit; IMV/ECMO, invasive mechanical ventilation/extracorporeal membrane oxygenation; IPTW, inverse probability of treatment weighting; LFO, low flow oxygen; mono, monotherapy; NSOc, no supplemental oxygen charges; RDV, remdesivir; SMD, standardized mean difference; SNF, skilled nursing facility. | | | | | | | |

**Figure S1.** 14- and 28-day mortality in patients hospitalized for COVID-19 receiving remdesivir + dexamethasone or dexamethasone monotherapy by supplemental oxygen requirements: Inverse probability of treatment weighting


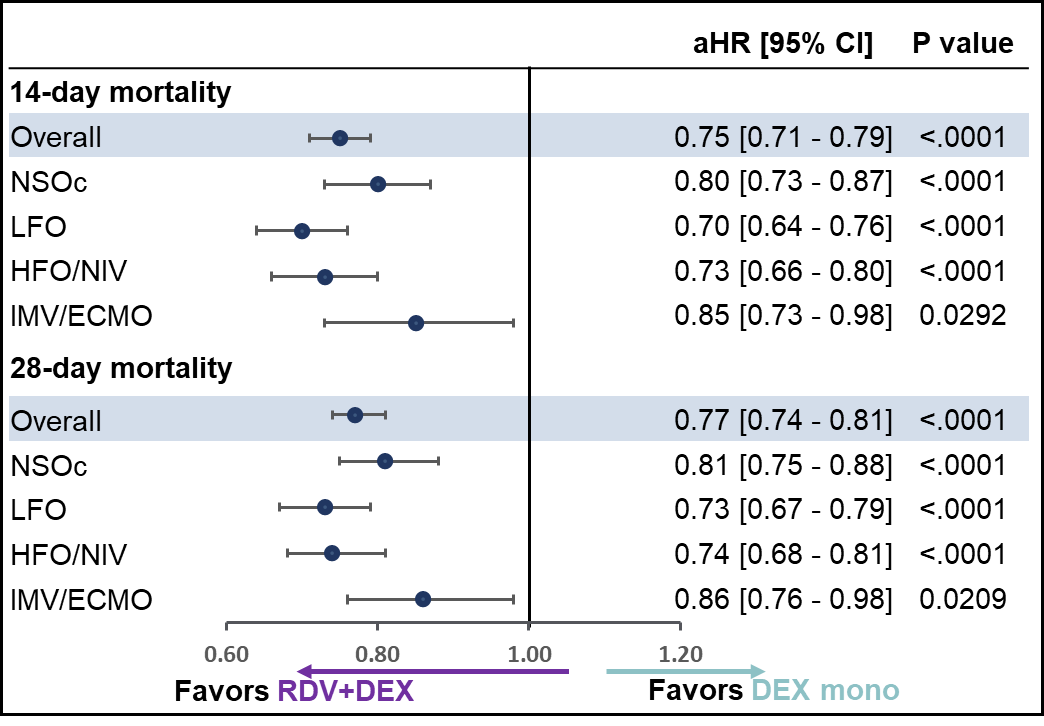


Estimates adjusted for age, admission month, hospital ward on admission (ICU vs. general ward, and time-varying treatment with other COVID-19 medications (baricitinib, tocilizumab, oral antivirals)
Abbreviations: aHR, adjusted hazard ratio; CI, confidence interval; COVID-19, coronavirus disease 2019; DEX, dexamethasone; HFO/NIV, high flow oxygen/non-invasive ventilation; ICU, intensive care unit; IMV/ECMO, invasive mechanical ventilation/extracorporeal membrane oxygenation; mono, monotherapy; LFO, low flow oxygen; mono, monotherapy; mono, monotherapy; NSOc, no supplemental oxygen charges; RDV, remdesivir.

**
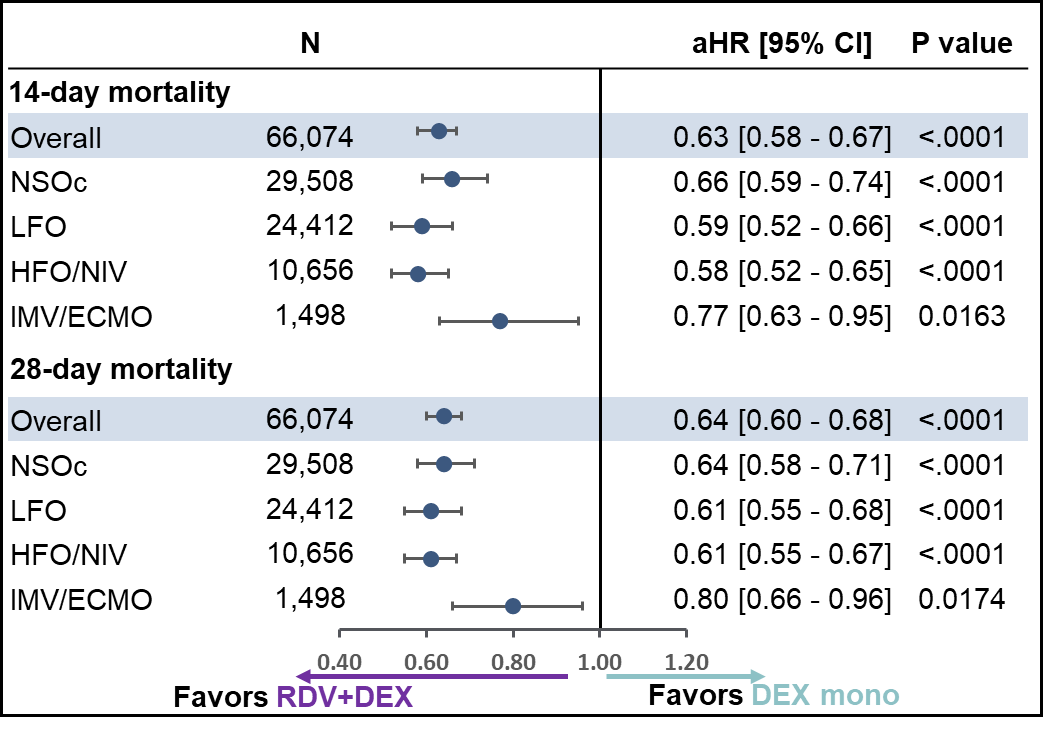
Figure S2.** 14- and 28-day mortality in patients hospitalized for COVID-19 receiving remdesivir + dexamethasone or dexamethasone monotherapy by supplemental oxygen requirements: 1:1 propensity score matching without replacement (outcome = expired only)

Estimates adjusted for age, admission month, hospital ward on admission (ICU vs. general ward, and time-varying treatment with other COVID-19 medications (baricitinib, tocilizumab, oral antivirals)
Abbreviations: aHR, adjusted hazard ratio; CI, confidence interval; COVID-19, coronavirus disease 2019; DEX, dexamethasone; mono, monotherapy; HFO/NIV, high flow oxygen/non-invasive ventilation; ICU, intensive care unit; IMV/ECMO, invasive mechanical ventilation/extracorporeal membrane oxygenation; mono, monotherapy; LFO, low flow oxygen; mono, monotherapy; NSOc, no supplemental oxygen charges; RDV, remdesivir.


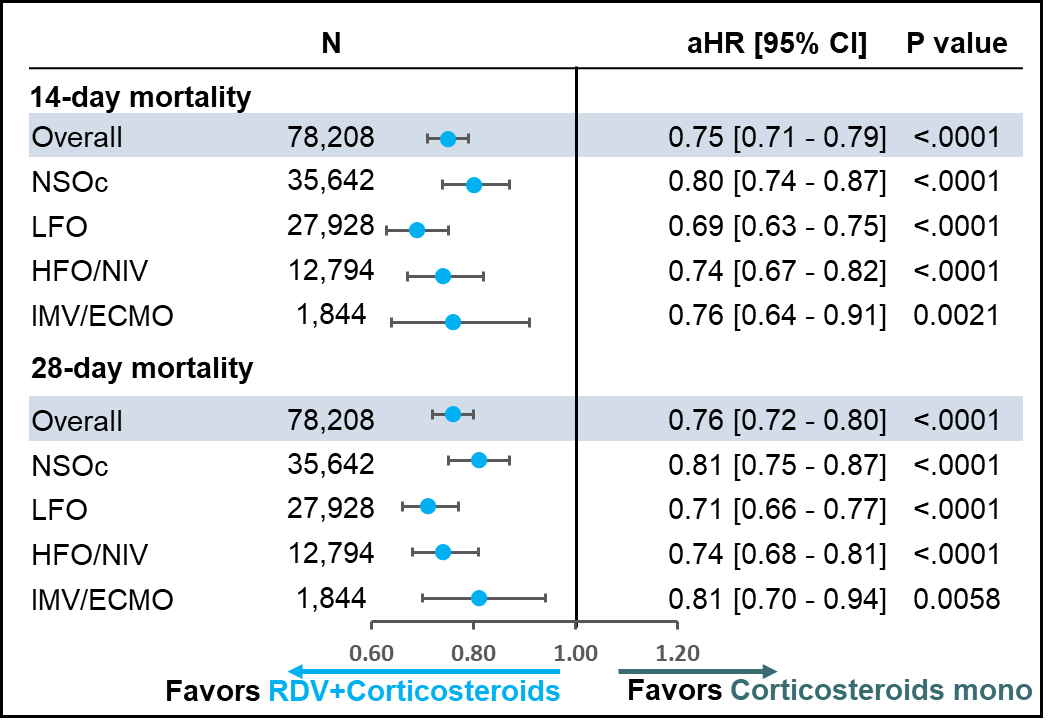
**Figure S3**. 14- and 28-day mortality in patients hospitalized for COVID-19 receiving remdesivir + corticosteroids or corticosteroid monotherapy by supplemental oxygen requirements: 1:1 propensity score matching without replacement

Estimates adjusted for age, admission month, hospital ward on admission (ICU vs. general ward, and time-varying treatment with other COVID-19 medications (baricitinib, tocilizumab, oral antivirals)
Abbreviations: aHR, adjusted hazard ratio; CI, confidence interval; COVID-19, coronavirus disease 2019; HFO/NIV, high flow oxygen/non-invasive ventilation; ICU, intensive care unit; IMV/ECMO, invasive mechanical ventilation/extracorporeal membrane oxygenation; LFO, low flow oxygen; mono, monotherapy; NSOc, no supplemental oxygen charges; RDV, remdesivir.
